# Practical Experiences of a Smart Livestock Location Monitoring System Leveraging GNSS, LoRaWAN and Cloud Services

**DOI:** 10.3390/s22010273

**Published:** 2021-12-30

**Authors:** Mike O. Ojo, Irene Viola, Mario Baratta, Stefano Giordano

**Affiliations:** 1 Department of Veterinary Sciences, University of Turin, 10095 Grugliasco (TO), Italy; irene.viola@unito.it (I.V.); or mario.baratta@unipr.it (M.B.); 2 Department of Chemistry, Life Sciences and Environmental Sustainability, University of Parma, 43124 Parma, Italy; 3 Department of Information Engineering, University of Pisa, 56126 Pisa, Italy; stefano.giordano@unipi.it

**Keywords:** livestock monitoring, smart agriculture, AWS architecture, cloud computing, LoRaWAN

## Abstract

Livestock farming is, in most cases in Europe, unsupervised, thus making it difficult to ensure adequate control of the position of the animals for the improvement of animal welfare. In addition, the geographical areas involved in livestock grazing usually have difficult access with harsh orography and lack of communications infrastructure, thus the need to provide a low-power livestock localization and monitoring system is of paramount importance, which is crucial not for a sustainable agriculture, but also for the protection of native breeds and meats thanks to their controlled supervision. In this context, this work presents an Internet of things (IoT)-based system integrating low-power wide area (LPWA) technology, cloud, and virtualization services to provide real-time livestock location monitoring. Taking into account the constraints coming from the environment in terms of energy supply and network connectivity, our proposed system is based on a wearable device equipped with inertial sensors, Global Positioning System (GPS) receiver, and LoRaWAN transceiver, which can provide a satisfactory compromise between performance, cost, and energy consumption. At first, this article provides the state-of-the-art localization techniques and technologies applied to smart livestock. Then, we proceed to provide the hardware and firmware co-design to achieve very low energy consumption, thus providing a significant positive impact to the battery life. The proposed platform has been evaluated in a pilot test in the northern part of Italy, evaluating different configurations in terms of sampling period, experimental duration, and number of devices. The results are analyzed and discussed for packet delivery ratio, energy consumption, localization accuracy, battery discharge measurement, and delay.

## 1. Introduction

To reach the goal of having sustainable and intelligent industrial agriculture, smart agriculture is taking advantage of the Internet of Things (IoT) paradigm, artificial intelligence (AI), and Big Data, with cloud and virtualization technologies, to facilitate innovative applications which can be achieved through the provision of service and product development, real-time monitoring and diagnostics, processing, and distribution to consumer experience [1,2]. At the same time, there is a growing need to improve precision farming so as to optimize animal production and reduce approximations in the assessment of animal health. Engineering advances and decreasing costs of the new electronic technologies have allowed the development of many sensor-based solutions for the livestock industry [3]. These sensors are able to collect data automatically and in real time, enabling the early detection of specific problems (e.g., production loss, poor health, and threats to wellbeing) at group or individual level [4]. This technological approach is currently known as precision livestock farming (PLF). Sensing solutions are implemented in PLF systems at the level of the smallest manageable production unit, the “sensor-based individual animal” approach [5].

However, an important criticality must be taken into consideration, especially for supply chains operating in marginal rural areas where poor technological infrastructures and unreliable services (electricity, telephone, and internet networks) are available in many small ruminant farms (often located in mountainous and remote areas), reducing the development of new local initiatives and businesses based on PLF implementation [6,7]. As a further step of automation, sensors used in PLF systems should ideally be integrated with processing (such as artificial intelligence functions) and storage solutions that will provide a platform developed on a “cloud continuum” starting from the back-end of the large data center (hyperscale service provider) and dynamically providing virtualized functions even at the peripheral part of the network.

Over the centuries, pastoralism and transhumance (seasonal movement of livestock between grazing areas) created a wide variety of specific cultural landscapes. In general, especially in Europe, grazing is of particular importance for the preservation of open landscapes in the mountainous areas. For example, extensive grazing is considered vital for maintaining many biodiversity-rich habitats in Europe [8]. Livestock farming is, in most cases in Europe, unsupervised, thus making livestock localization and monitoring a relevant task in smart agriculture, which is crucial for a sustainable agriculture [9].

Livestock such as sheep are free in large enclosed remote areas for most of the year, although they are controlled periodically for veterinary control. Livestock localization helps to ensure adequate control of the position of the animals for the improvement of animal welfare, thus simplifying the everyday work of farmers and veterinary doctors. In addition, the supervision of livestock enhances welfare control processes, avoiding unnecessary casualties and allowing a more efficient reproductive process. It also provides a better management of available pastures [10]. Location information over time provides information about animal activity (i.e., the health status, etc.). For example, in the work of [11,12], changes in the activity may signal diseases, and can be often detected before emergence of clinical symptoms. Livestock localization and monitoring can be divided into two parts, which are the livestock location monitoring and livestock behavioral/activity monitoring [13]. This paper only focuses on the livestock location monitoring, but with the aim of adopting the cloud services that could be easily extended to provide new solutions in the specific domain (livestock behavioral/activity monitoring is out of the scope of this work).

Since the emergence of Global Navigation Satellite Systems (GNSS) solutions, such as Global Positioning System (GPS), GLONASS, or Galileo, the task of determining one’s location has become easy and smooth. However, this comes at the expense of high energy consumption from the GNSS receivers, but with highly accurate location estimations. There are other alternatives to GNSS for livestock localization, such as NB-IoT, LoRaWAN, etc., where accuracy seems to be an issue. Although the alternatives present low power consumption, these deployments are heavily dependent on the infrastructure (gateway installation might be difficult in the remote areas). Enabling localization in an IoT system is a trade-off between constraints, desired parameters, and functionalities of the system. This includes the aforementioned costs and the resulting localization accuracy.

Knowing the location data in (near) real time means that the device is equipped with a wireless transceiver. Communication technologies such as cellular communication are not suitable due to the high power consumption of cellular receivers, which is a major drawback for embedded devices today. In addition, radio coverage of cellular communication such as 3G, 4G, and 5G is not guaranteed everywhere, especially in the mountainous areas where telecommunication service providers find these places less interesting due to low population density, so alternate wireless communication is required. As the coverage of telecommunication infrastructures in rural and mountainous areas is generally poor or absent, and troubleshooting or maintenance operations are very difficult, we identified LoRa and LoRaWAN, a low-cost, low-power technology, as the most suitable connectivity solutions, especially for monitoring and control operations. In fact, as LoRaWAN is a low-power and long-range communication protocol—as will be further discussed in Section 2.1.4, it can provide connectivity over large grazing areas, also ensuring robustness with low energy requirements.

This paper is motivated by the Smartsheep project (https://www.smartsheep.it/, accessed on 20 October 2021) which aims at developing new biosensors in the control of animal health and the movements of a flock in the mountain pastures. In this context, this work presents an IoT-based localization system to provide real-time livestock location monitoring. The system integrates ad hoc IoT devices, LPWAN technology, and cloud computing. The ad hoc IoT device in wearable form is equipped with inertial sensors, GPS, and LoRaWAN transceiver. The device periodically collects location and activity data and then transmits it via the LoRaWAN gateway to the cloud system for storage, processing, and management. This work also presents a monitoring platform for enabling the remote monitoring and control of the IoT devices, which was carried out using virtualization technologies. In our work, we made use of Amazon Web Services (AWS), which is a scalable cloud-based architecture for a modular IoT system.

This approach is smart as it envisions that the development of an integrated infrastructure was not only computing, communications, and storage capabilities that will be integrated, but also, data moving towards the data center where cloudlets will be set up, scaled, and torn down to process the data in a more distributed, robust, and secure environment. This work is a starting point for the design of IoT platforms integrated with the functions provided by the new paradigms of cloud computing, such as edge or fog computing, integrating within the adoption of LoRaWAN and GPS a data-driven method, aimed to save time, cost, and also improving the process of improving humans’ quality of life in the domain.

The main contributions of this article are as follows:

1. A comprehensive state-of-the-art section is provided on the techniques and technologies used for livestock localization.
2. A description of the hardware design and the firmware used.
3. A detailed description of the design and development of the cloud-based monitoring platform for the livestock localization system consisting of the sensors, communication technology, and data-processing modules.
4. We discuss the results of experiments to evaluate the transmission quality of our testbed under various scenarios.

The rest of this paper is organized as follows: Section 2 introduces the state-of-the-art and the related works. The overall system architecture is described in Section 3. In Section 4, we present the experimental setup and the experimental results. Section 5 presents the discussion, and lastly, Section 6 concludes the paper with some final remarks.

## 2. State-of-the-Art and Related Works

In this section, we briefly discuss the state-of-the-art techniques and technologies used in livestock localization. Next, we present the related works.

### 2.1. Livestock Localization

**Figure 1:**
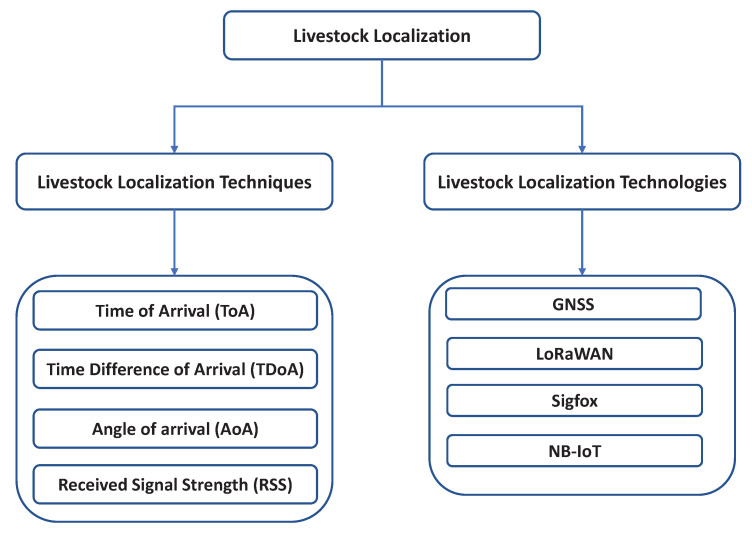
Livestock localization taxonomy.

The essential features of a successful localization scheme in sheep farming are small size, energy-efficiency management, low cost, due to the potentially high number of nodes required for monitoring an entire herd of sheep, synchronization time, to obtain a reasonable operation time to acquire the device’s location, appearance, to equip these devices with the appropriate level of protection, e.g., waterproof protection, and independence from additional hardware which can increase the costs and reduce mobility. Livestock localization is an essential process in the IoT environment for tracking and monitoring livestock with the help of sensor nodes. The sensor nodes collect the target information and transfer it to the central controller for further processing. These applications demand information about the position of the sensor node. Node localization algorithms are mainly categorized as range-based localization and range-free localization. In the range-based method, the node locations are estimated by considering point-to-point distance or angle between the nodes with some reference, whereas the the range-free method is based on the connectivity or pattern mapping for location approximation. In this work, we are only focusing on range-based methods which are usually used in livestock localization. They utilize hop distances, hop counts, and angles for a position estimate. We classify livestock localization into its techniques and technologies. The most widely used techniques for geolocation of wireless end devices are based on the measurement of certain parameters (e.g., signal attenuation, signal propagation time, and angle) by other devices (e.g., the GWs) with a known location. The taxonomy of livestock localization is illustrated in Figure 1. The basic methods of geolocation include [14] the following:

- **Time-of-Arrival (ToA)** [15,16] utilizes the signal propagation time to calculate the distance between the transmitter and the receiver through the use of synchronized clocks. ToA uses time stamps labeled in the transmitted signals along with the received time to determine the distance the signal had traveled. ToA is one of the most accurate techniques available, but a perfect synchronization between the transmitters and receivers is important, thus also introducing complexity to the system. The key factors that affect ToA estimation accuracy are the signal bandwidth and the sampling rate. Low sampling rate (in time) reduces the ToA resolution as the signal may arrive between the sampled intervals. Frequency domain super-resolution techniques are commonly used to obtain the ToA with high resolution from the channel frequency response. In addition, in a TOA-based localization system, devices in the network need synchronized clocks, which require additional hardware, thus increasing the cost of the system.
- **Time-Difference-of-Arrival (TDoA)** measures the difference of propagation time between the signals in terms of their nature, such as using RF, acoustic, or ultrasonic signals [17]. The idea is that the distance is calculated by determining the differences in arrival time of the packet to the different receivers. This method is affected by delay that can be experienced by the transmitted signal, as the different distances are calculated based on the propagation times. This method sometimes controls the problem synchronization, and also reduces complexity [18].
- **Received Signal Strength Indicator (RSSI)** has gained much attention in the last years [19] due to the increasing number of IoT devices utilizing these methods for localization. RSSI measurements are commonly used for target detection, but one can also use them for localization without any additional sensor functionalities. RSSI utilizes some signal propagation models, either theoretical or empirical, to translate signal strength into distance. The received signal strength measurement is also highly sensitive to the interference and may experience significant deviations from one measurement to another.
- **Angle-of-Arrival (AoA)** is based on angle calculation of which direction the signal is received from (i.e., sent by the node) [20]. AoA systems use an array of antennas to determine the angle from which the signal is propagated. Triangulation is then performed, along with the geometric angles of triangles, to determine the position of the receiver. Using AoA techniques to estimate a position does not require time synchronization between the measuring units, and the position can be determined with as few as three measuring units for 3D positioning or two measuring units for 2D positioning. AoA techniques come at the price of requiring complex hardware and must be calibrated in order to obtain an accurate position [21].

Table 1 provides the pros and cons of the discussed techniques for livestock localization.

In this section, several existing technologies which have been used to provide livestock localization services will be presented, as well as their mode of operation. Radio communication technologies such as LoRa, Narrowband Internet of Things (NB-IoT), Sigfox, and GNSS will be presented first, followed by other radio technologies such as Bluetooth Low Energy (BLE) and Zigbee technology, which are meant for indoor localization services but few works have been seen to use it for livestock localization.

#### 2.1.1. NB-IoT

Narrowband Internet of Things (NB-IoT), proposed by 3rd Generation Partnership Project (3GPP), is a variant of 4G Long Term Evolution (LTE) developed to fulfill the IoT requirements in low data rate applications. It is also known as LTE Cat-NB1 and operates in the licensed spectrum. It belongs to the LPWA technologies, which could work virtually anywhere when the infrastructure is present. It supports three different modes of operation, namely standalone operation (as a dedicated carrier), in-band operation (deployed within an LTE wide-band system), and lastly guard-band operations (co-located with an LTE cell) [22]. NB-IoT is also being used in many commercial agricultural solutions. The implementation of NB-IoT is only possible through telecommunication providers’ IoT services. Therefore, it is neither cost effective, nor does it provide openness as offered by other IoT technologies.

In the 3GPP Rel-13 and 3GPP Rel-14 of NB-IoT, localization can be achieved through the use of enhanced cell ID (eCID) [23]. This method allows the NB-IoT user to measure and report the enhanced Node-B’s (eNB’s) receiver–sender time difference, reference signal received power (RSRP), and the reference signal received quality. The 3GPP Rel-14 also introduced observed time difference of arrival (OTDOA), another method for determining the user’s position. OTDOA introduces a new narrowband positioning reference signal (NPRS), which is sent to the receiver to enhance the positioning measurement. In OTDOA, the ToA of NPRS from a reference eNB and the neighbour eNBs are estimated. By measuring the time difference, the user’s reference signal time difference (RSTD) can be estimated. Each RSTD measurement restricts the user’s position to a hyperbola. The point of intersection of several such hyperbolas give the user’s location [24]. There are still ongoing works on the design and features of NB-IoT on how to improve the localization accuracy [25].

#### 2.1.2. GNSS

Global Navigation Satellite Systems (GNSS) solutions, such as GPS, GLONASS, GALILEO, and BeiDou, are the most common localization systems used for outdoor localization purposes, such as locating and tracking humans, cars, livestock, and assets, among others [26]. GNSS systems provide accurate location estimations compared to other technologies, especially where the satellites are directly visible, i.e., having line of sight (LoS), but at the expense of high energy consumption.

The working principles of any GNSS satellites are almost the same. The satellites broadcast a very precise timing signal and data message, called navigation message, that contains their orbital parameters. GNSS receivers receive the navigation message sent from the relevant satellites in orbit, process the message, and estimate position velocity and time. A minimum of four satellites are needed for estimating three-dimensional position and time. At first, the receiver calculates its distance from each visible satellite and then calculates a three-dimensional position using the trilateration or multitrilateration technique. Good accuracy can be obtained if visible satellites are broadly spaced in the sky. The synchronization of the receiver clock and the satellite clocks are very crucial. The satellites carry atomic clocks onboard, which makes their timing very precise [26,27].

#### 2.1.3. Sigfox

Sigfox is an LPWAN technology, highly efficient in spectrum usage. Sigfox, an LPWAN network operator, deploys its proprietary base stations equipped with cognitive software-defined radios and connect them to the back-end servers using an IP-based network. It utilizes the ultra narrow band carrier of the sub 1 GHz ISM bands and binary phase shift keying (BPSK) modulation technique. It uses star topology, and the base station is equipped with a cognitive software-defined radio that is connected to servers using an IP-based network. The communication range of Sigfox is up to 45 km and 12 km in rural and urban areas, respectively. Sigfox supports data rates of up to 250 kbps, and also uses unlicensed spectrum (868 MHz and 902 MHz) for communications. For each end-node, Sigfox restricts downlink communications to 4 transmissions of 8 bytes of payload, and uplink communications to 140 transmissions of 12 bytes of payload [28]. Despite these limitations, Sigfox provides many opportunities in smart agriculture [29]. Sigfox presents its own localization feature, which is based on the RSSI coming from the messages sent by a device and received by the base stations of the Sigfox infrastructure combined with machine learning algorithms [30]. This method is calculated using regular Sigfox messages, which is very cost-efficient with no extra hardware required, and the message payload can be empty (less battery use) or used for regular handling. Radio choices made by Sigfox bring specific benefits: low energy for a longer battery life, low connectivity rate, high network capacity, long range, and resilience to interference, with the capacity to resist jamming. Access to the service strongly depends on the Sigfox network coverage in the dedicated territory, which is a major limitation.

#### 2.1.4. LoRa/LoRaWAN

LoRa and LoRaWAN are global de facto standards of low-power wide-area network (LPWAN), and they are the most adopted technologies for the IoT. LoRa, the physical layer in LoRaWAN, uses forward error correction (FEC) and a proprietary modulation which is a variant of chirp spread spectrum (CSS) [31]. The physical channel is logically separated by the spreading factor (SF) due to its orthogonality. The carrier frequency varies over a designated amount of time, thus achieving low power, robustness, and long-range communication links [32]. LoRa is defined by its main parameters (SF, bandwidth (BW), and code rate (CR)), which are configured to adapt to the working scenario. It is worth noting that different combinations of the aforementioned transmission parameters yield different trade-offs with respect to the range and data rate that can be achieved, and these combinations are also governed by regulatory constraints. A network structure based on LoRaWAN protocol consists of four individual sections, namely, the end devices, the gateway, the network server, and the application server. The LoRaWAN MAC layer provides the medium access control mechanism operating as ALOHA protocol, which enables communication between multiple devices and network gateway(s). Given the features of LoRa such as long range and low power, several agricultural applications [33,34,35,36] have been developed where LoRaWAN has been exploited for controlling and monitoring.

RSSI for coarse positioning and TDOA for finer accuracy are the two LoRaWAN protocol methods for geolocation [37]. The LoRa Alliance claims that using TDOA in LoRaWAN has an accuracy range of 20–200 m depending on conditions, whereas in RSSI, it is about 1000–2000 m accuracy. Janssen et al. evaluated an RSS fingerprint-based LoRaWAN method using a random forest machine learning algorithm where they were able to obtain an average location estimation error of 340 m [38]. Further research has to be conducted to improve the location accuracy in LoRaWAN localization.

Bluetooth Low Energy (BLE) and Zigbee technology are low-power, low-cost wireless systems used in agricultural applications. BLE merged into the main Bluetooth standard in July 2010, when Bluetooth Core Specification 4.0 included the classic Bluetooth protocol, Bluetooth High-Speed Protocol, and BLE [39]. BLE is designed for very low-power applications that can run off a coin cell battery for months or even years. BLE has been subsequently enhanced in versions of BLE 5 by addressing inadequacies via the implementation of pure mesh topology to provide enhanced network coverage, internetwork connectivity, and improved security [40]. BLE can be compared to other wireless technologies such as 4G/5G, Wi-Fi, and LPWAN technologies with the following features. Firstly, considering privacy, in the case of BLE, the Bluetooth facility needs to be switched on to allow location detection. BLE allows privacy and freedom in terms of sharing data in public compared to some wireless technology such as Wi-Fi. Secondly, in terms of speed, BLE is better for transmitting smaller amounts of data such as sensor readings of temperature, GPS coordinates, and acceleration details, which is ideal in the case of agricultural spectrum. Thirdly, BLE can also be used for localization, which is usually carried out by installing a set of proximity beacons (i.e., BLE transmitters) at known locations [19]. Receivers extract the RSSI (which is a proxy of the distance from the transmitter) from the nearest beacons and use these values to predict their own position. Despite the several uses of BLE-localization in indoor environments, BLE-localization poses a lot of challenges in the industrial and agricultural environments, especially outdoor environments, because of the harsh conditions and environments. Despite these challenges, few works [41,42] have utilized BLE for livestock localization.

Zigbee is built upon the IEEE 802.15.4 standard that is concerned with the physical and MAC layers for low cost, low data rate, and energy-efficient personal area networks [43]. ZigBee is favorable for localization of sensors in wireless sensor networks (WSN), but it is not readily available on the majority of the devices, hence it is not favorable for livestock localization. Few works, such as [44,45], have utilized Zigbee technology for livestock monitoring.

### 2.2. Related Works

The smart agriculture concept, with regards to localization, relates to location-aware devices to monitor the movement of animals and raise alerts when they violate the boundary of the geofence of the farm or pasture. There have been several studies on the integration of GNSS technology with cellular communication for applications regarding livestock location monitoring [46]. GPS technology occupies the majority of the deployment in the literature compared to other localization technologies. This is hardly surprising given the easy adoption and accurate location estimations compared to other technologies, especially where the satellites are directly visible. In addition, GNSS technology has achieved a lot of success in detecting static or dynamic unitary behaviors differentiated through changes in path speeds: foraging or grazing, resting, and walking [47]. The authors in [48] presented a data collection collar for vital signs of cattle on the grassland based on GPS and LoRa technology. Similar work using GPS technology is also addressed in [49].

Livestock theft management is one of several use cases in livestock location monitoring. To this end, researchers have proposed several systems that can be used to minimize the chances of livestock theft. In [50], the authors proposed a system based on RFID and GPRS technology for tagging, identification, and communication, which is supported by a centralized database system. The authors introduced an approach to identify an animal stolen if the animal is found in a geographic location that is considered far from the registered location of the animal. Similar work addressing livestock theft management is addressed in [51].

Few works have addressed the use of other technologies, such as BLE and LPWAN, for livestock location monitoring. In [41], a BLE technology with RSSI localization method was designed for livestock location monitoring. The method introduced by the authors provided accurate localization using a small number of reference points (anchors) and required limited measurements during setup. Zigbee was also used in [44], where the authors presented a localization scheme in wireless sensor networks for cattle monitoring applications in grazing fields, where the use of link-quality indicator-based ratiometric vector iteration (RVI) algorithm was utilized. Similar studies utilizing Zigbee technology for livestock monitoring have been demonstrated in [45,52]. To the best of our knowledge, several studies on livestock location monitoring implementations use GNSS receivers to send GPS coordinates over LPWAN, such as in LoRaWAN [53,54], NB-IoT [55], and Sigfox [56].

Another alternative for livestock monitoring is the use of unmanned aerial vehicles (UAVs) as described in [57,58,59]. Some of the application uses of UAVs do not explore the imaging capabilities of the UAV, rather using the aircraft for different actions, and collection of data from sensors fitted on the animals through wireless communication [60,61]. For example, the authors in [62] combined the usage of GNSS and UAVs for livestock monitoring, where drones were deployed to accomplish the sweep coverage of the entire pasture, and to determine the tracking information acquired by GPS collars won by the animals. Other uses of UAVs for livestock monitoring involve exploring images for direct visual analysis, aiming at cattle detection [63] and determination of feeding behaviour [64]. However, there are some limitations and technical issues when using UAVs in agriculture, especially for livestock monitoring, such as payload and battery capacity, cost, environmental factors, and operational factors where special permissions are needed in some countries [65]. Lastly, in addition to the related works on livestock localization, there are several works [66,67,68,69] that have applied AWS cloud services to agriculture.

In summary, whilst the surveyed studies are focusing on the performance of GNSS technology for livestock localization, more studies on practical implementations of using GNSS and LPWAN technology need to be carried out on livestock localization. Such experimental implementations over a period of time are needed to provide an insight on accuracy and energy consumption. Table 2 presents a brief summary of the deployments for livestock localization.

## 3. System Architecture

**Figure 2:**
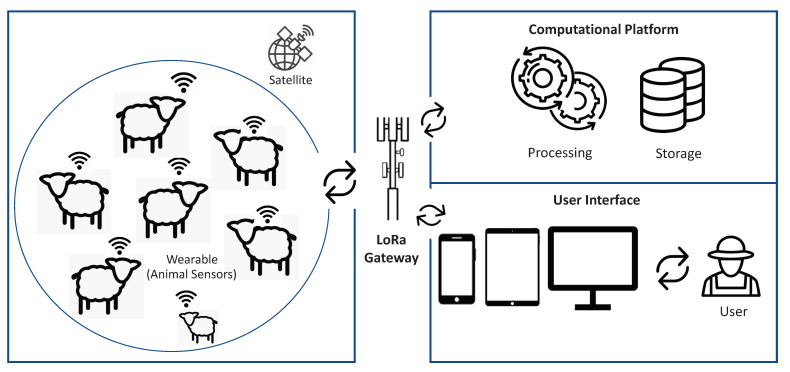
System architecture.

In this section, the overall system architecture is presented (see Figure 2). Next, the mode of operation of the device and the system infrastructure are also described.

### 3.1. Device Description

**Figure 3:**
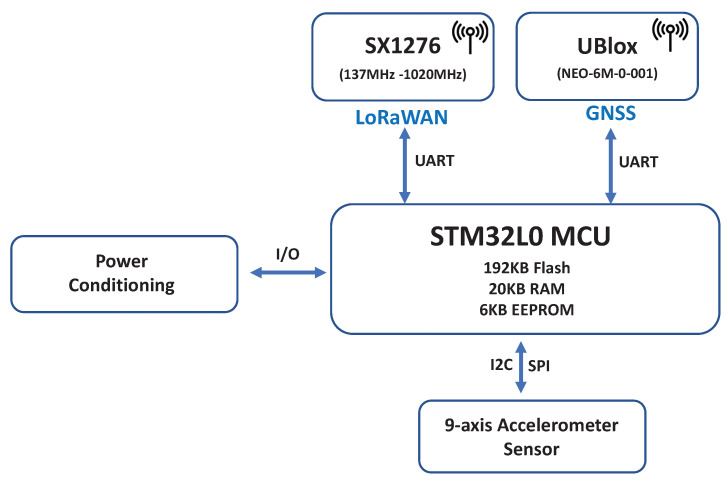
Hardware block diagram.

The end device is built around an STM32L072 [71], a 32-bit ARM Cortex^®^ M0+ core, which combines a 192 KB flash memory with read-while-write capabilities, 6 kB EEPROM, 20 kB SRAM, general purpose I/O lines, and peripheral communication interfaces (USART, $I^{2}C$, SPI bus). It also features a GPS positioning module and an LoRa transceiver able to transmit using FSK and LoRa modulations for communication purposes. The end device embeds an sx1276 module supported by LoRaWAN and also integrates a nine-axis accelerometer (three-axis gyroscope, three-axis accelerometer, and three-axis magnetometer) necessary to provide information about accelerations in all three directions and rotations around each axis. The hardware block diagram is shown in Figure 3. The LoRaWAN transceiver was configured as a device of class A, which is necessary to ensure minimum energy consumption of the device. To transmit, process, and store the information retrieved from the devices, we used a proxy software that collects and transmits this information via LoRa first to the LoRaWAN gateway, and then to the back-end system.

### 3.2. Mode of Operation

In this work, we configure the device to adapt to the detected motion of the device to improve the energy efficiency. At power on, the device works with the last-used settings. The settings (physical layer parameters, role, etc.) are stored in the non-volatile flash memory and reloaded at power-up. The device supports both ATP/OTAA methods to communicate to the LoRaWAN network. In our case, the device is configured with the OTAA registration keys, where the device will repeatedly try to join the LoRaWAN network until the join process is successful. Once the join process is successful, the device starts collecting data retrieved from the sensors to the LoRaWAN network.

In the data collection phase, the device will try to obtain the location information (i.e., latitude, longitude, altitude, hdop) from the satellite in a periodic manner. Other information retrieved from the sensors, such as battery, accelerometer, etc., are collated with the location information to be forwarded to the LoRaWAN gateway. If the location information is not determined within X seconds (configurable), the location information field is set to 0x00, while other information, such as the battery status, enters the uplink phase. In the uplink phase, the device sends all the messages retrieved from the sensors to the LoRaWAN network in a periodic manner. Once the messages are sent, the device enters into a sleep state to save power. Depending on how much time has passed since the last physical movement of the device, which is determined by the motion sensor of the device, the device enters different sleep states. The states are the active sleep phase and the passive sleep phase.

**Figure 4:**
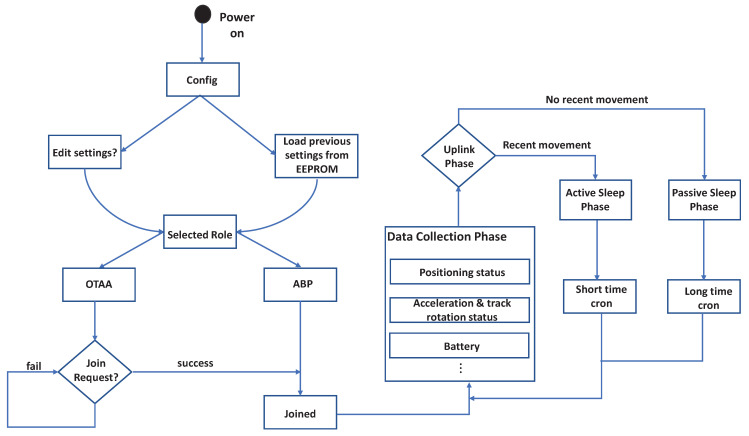
State machine diagram of the device.

In the active sleep phase, the device uploads its information to the LoRaWAN network, then enters the sleep state to conserve energy. The frequency at which the device wakes up in the active sleep phase (active mode) can be configured using a cron expression. In our application, typical values are between 10 min or 15 min. When the device is in the active sleep phase, the device will not be triggered to gather more location information through motion, but the movement of the device will still be registered to keep the device in the active mode. In the passive sleep state, if no motion is detected for a long amount of time, the device enters a passive sleep state, but the device will be activated through movement. Typical values for the cron in the passive sleep phase in our application are between 90 min or 120 min. If no physical movement is detected at the end of the long time cron, the device sends the last-used location information along with other information to the LoRaWAN network, whereas if a physical movement is detected by the internal motion sensor during this phase, the device immediately wakes up and switches to active mode. A summary of the operating logic of the device is presented with the simplified state machine diagram in Figure 4.

### 3.3. System Infrastructure

There is a need to use cloud solutions to ensure a reliable and secure infrastructure that supports automatic scaling of resources according to the system needs. The use of cloud services leads to increased scalability, availability, reliability, agility, and security, among others, where there is a huge advantage of moving the IT infrastructure to specialized cloud providers [72]. We made use of AWS as the cloud infrastructure due to its numerous services provided for IoT applications [73]. AWS is one of the five major solutions with the largest market share alongside equivalent IoT platforms from Microsoft, Cisco, Google, and IBM [74].

**Figure 5:**
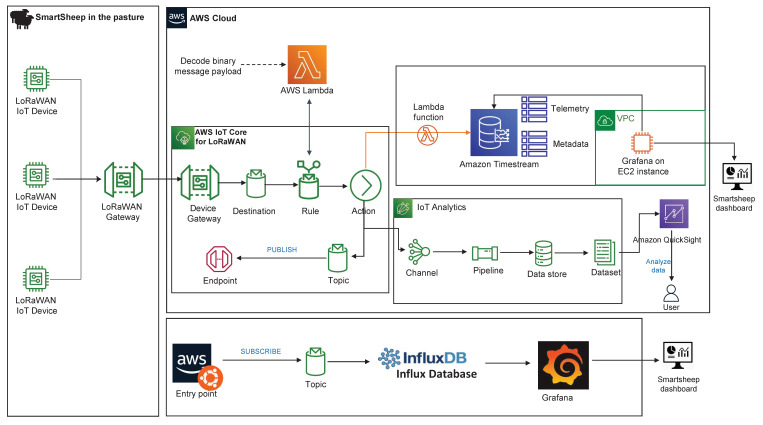
Smartsheep system infrastructure and communication flow.

AWS Cloud contains many groups of services, including IoT Core, IoT Core for LoRaWAN, compute, storage, databases, network, management, application, analytics, and others. The system infrastructure and the services used in our Smartsheep system are illustrated in Figure 5. The IoT devices and the LoRaWAN gateway used in the Smartsheep system are registered in the AWS IoT Core to connect to the AWS cloud, without developing or operating an LoRaWAN Network Server (LNS). In order for the IoT devices developed for the Smartsheep to interact with the AWS cloud services, an IoT rule is needed. The IoT rule has so many functions, such as filtering data coming from the IoT devices, sending the LoRaWAN messages to the AWS Lambda to decode the payload, sending LoRaWAN messages to the database channel, analytics channel, etc.

Our application can be implemented in various deployment scenarios. In our case, two different deployment scenarios were devised to better analyze the resource consumption of our application when using cloud-based IoT services, as shown in Figure 5. In the first deployment scenario, AWS infrastructure was used in the entirety of the implementation. The raw messages gathered by the sensors are sent to AWS lambda to decode the messages using the rule function. The decoded messages with other information are archived into a channel, which stores all data from a certain MQTT topic. The MQTT protocol was created especially for low latency and small-sized packets characteristic of IoT devices. Messages from the channel can be redirected by the IoT Core to AWS IoT Analytics by means of user-defined rules. The dataset is imported into AWS QuickSight for graphical representation. Furthermore, the messages from the IoT Core, decoded by the AWS lambda layer, are written by the lambda function as measures into the Amazon Timestream table (telemetry and metadata). Amazon Timestream is a fast, scalable, and serverless time series database for IoT applications. The data from the Amazon Timestream are displayed in Grafana. The main advantage of using this deployment scenario is that the serverless infrastructure is managed entirely by AWS and, as such, this solution requires the least time and effort to be deployed.

In the second deployment scenario, the data gathered from the devices registered on AWS IoT core for LoRaWAN were sent to InfluxDB and Grafana containers, which store and display data for each device. The docker containers, which contain InfluxDB, Grafana, and other applications, are installed on a local server. The monitoring dashboard provided by InfluxDB and Grafana offers a useful insight to drive the experiment without high maintenance cost.

## 4. Results

### 4.1. Experimental Testbed and Configurations

**Figure 6:**
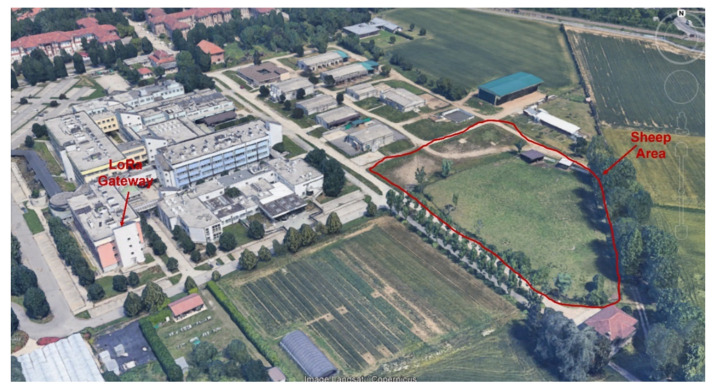
LoRaWAN gateway and the sheep area.

**Figure 7:**
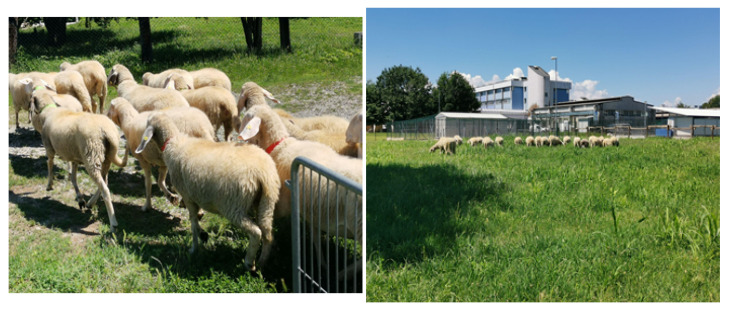
A flock of sheep grazing in a field.

We conducted a pilot test to assess the effectiveness of the Smartsheep location monitoring system. The test environment is located at the pasture area of the Department of Veterinary Sciences, Grugliasco, University of Turin, Italy, as shown in Figure 6. The approximate area where the flock of sheep were localized is approximately 700 m${}^{2}$. The flock of sheep grazed freely throughout the experimental test. All sheep selected for each experiment carried an end-device collar for localization, as shown in Figure 6. The devices transmit information (latitude, longitude, battery, etc.) via LoRa to the LoRaWAN gateway, and then to the back-end system for visualization. The activity is transmitted from the devices to the LoRaWAN gateway in a periodic manner. We make use of a commercial gateway embedded with LoRa capabilities placed on top of a building at a height of 40 m, as shown in Figure 6. The LoRaWAN gateway is connected to an ADSL router that provides connectivity with the Internet and finally to the AWS network. BW and CR were kept constant for our field experiment (125 kHz, 4/5) while SF was varied between (7, 8, 9, 10, 11, 12) when the adaptive data rate (ADR) was disabled. To study the message frequency in the Smartsheep LoRaWAN network, we considered three communication schemes: sending packets every 5, 10, and 15 min. Ten end devices were considered for the pilot test. This is necessary to evaluate the performance of the battery discharge on the time activity of the devices. Figure 7 shows an image of a flock of sheep grazing in a field during the pilot test.

### 4.2. Convergence Time

**Figure 8:**
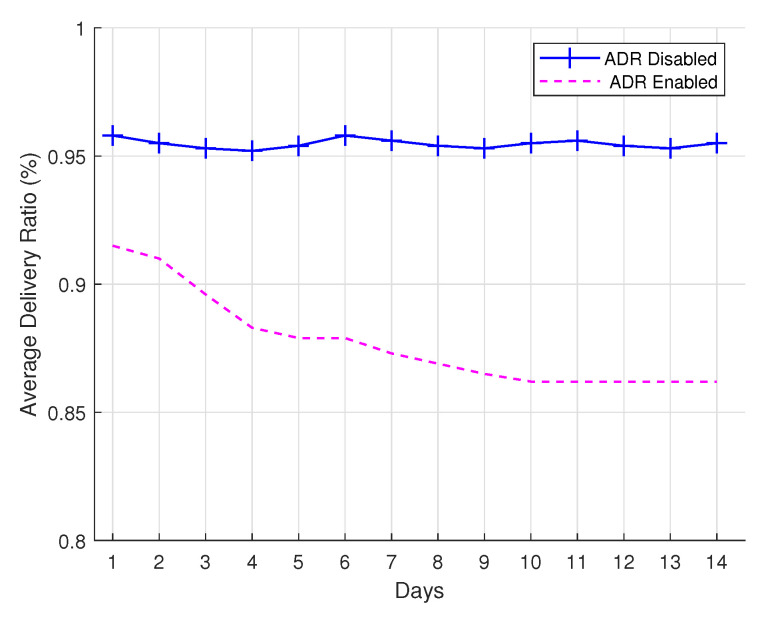
Convergence time.

In this subsection, we evaluate the performance of the LoRaWAN network with respect to convergence time. LoRaWAN networks can operate with or without the ADR. The ADR is a component that controls the performance of the LoRaWAN network by modifying the data rate parameter (i.e., spreading factor) based on current wireless conditions. This is important to reduce the overall energy consumption, and it increases the overall delivery ratio with the correct selection of transmission parameters, as demonstrated in [75,76]. In our work, we implemented the ADR technique presented in the LoRaWAN specifications. In Figure 8, we present the convergence time of the Smartsheep LoRaWAN network when the ADR is enabled/disabled. The LoRaWAN MAC layer has an optional ADR mechanism, if enabled, to ensure the end devices modify the chosen data rate based on the recent traffic conditions. The network server makes better decisions when more packets flow into the network. In this experiment, a total of 14 days evaluation is considered to assess the performance of the LoRaWAN network when the ADR is enabled/disabled at the network server. We consider 10 end devices that send their activity every 15 min to the LoRaWAN network. From the figure, we can observe that when the ADR is disabled, the delivery ratio tends to remain stable due to fewer changes in the network operation, whereas when the ADR is enabled, there are instabilities in certain metrics, such as delivery ratio. As the packets in the network increase in the ADR-enabled mechanism, the network server tends to have more data to make better decisions, thus making the metrics (i.e., delivery ratio) stabilize and converge as the communication time increases. The metrics can be considered more stable in the ADR-disabled mechanism compared to the ADR-enabled mechanism in a short time period, but as the number of days increases, both mechanisms tend to be stable.

### 4.3. Delivery Ratio

**Figure 9:**
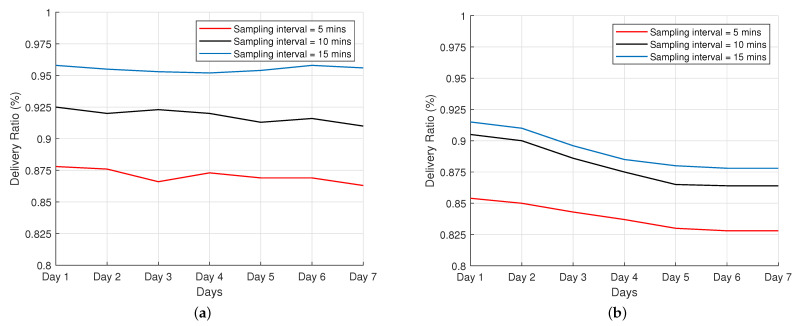
Average delivery ratio at the network server. (**a**) ADR disabled; (**b**) ADR enabled.

This subsection presents the results from the experimental field test regarding the packet delivery ratio (PDR). PDR is calculated as the number of packets received by the gateway to the total number of packets sent, with a value of 1 implying 100% success and a value of 0 implying no success at all. The result is shown in Figure 9. We consider three communication schemes: sending packets every 5, 10, and 15 min, and the test was performed for a 7-day period for both ADR-enabled scheme and ADR-disabled scheme. We can draw the following remarks: For a small number of nodes, which is relatively the situation we considered in this experiment, LoRa networks with ADR disabled achieve a better delivery ratio than those with ADR enabled. This situation can be explained in the following context: a small number of nodes implies a less-saturated spectrum and limited possible collisions. Under these circumstances, network ordering can be optional when deploying these networks in small areas.

LoRa networks achieve a better delivery ratio with an activity of 15 min as compared to 10 min and 5 min for both ADR-enabled and ADR-disabled schemes. When varying the sampling interval, all curves show a standard behavior corresponding to their interval: sampling at 5 min, which denotes more messages, implies a lower delivery ratio, as more nodes are transmitting simultaneously and occupy the same channel. Nonetheless, the results for both schemes are over 82.5%, which is expected for a network with these characteristics.

### 4.4. Energy Consumption

**Figure 10:**
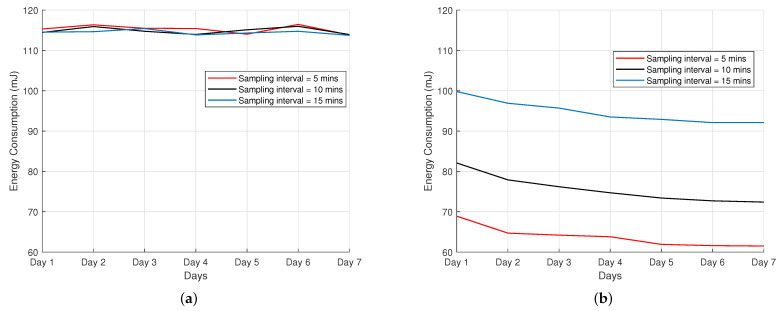
Energy consumption: (**a**) ADR disabled; (**b**) ADR enabled.

This subsection presents the assessment of the energy consumption for end devices obtained via field experiments by varying the packet frequency. The field test experiment was performed for a 7-day period for both ADR-enabled scheme and ADR-disabled scheme, where the results are shown in Figure 10a,b. It is essential to note that the energy consumption shown in the figures corresponds to the average energy consumption for the nodes in the network in millijoule (mJ).

It can be seen from Figure 10a that the energy consumption in the ADR-disabled scheme is almost invariant for the days considered when varying the sampling intervals. In the ADR-enabled scheme, the energy consumption of the devices performed better than the ADR-disabled scheme. We observe that when the ADR-enabled networks are considered, as shown in Figure 10b, the effect of transmission control is noticeable. When frequent transmissions occur in a network, this is the most energy-efficient scenario. The results show a substantial energy reduction when operating in the ADR scheme, especially when frequent communication from end devices to the network server is very frequent (every 5 min interval).

### 4.5. Battery Discharge Measurement

**Figure 11:**
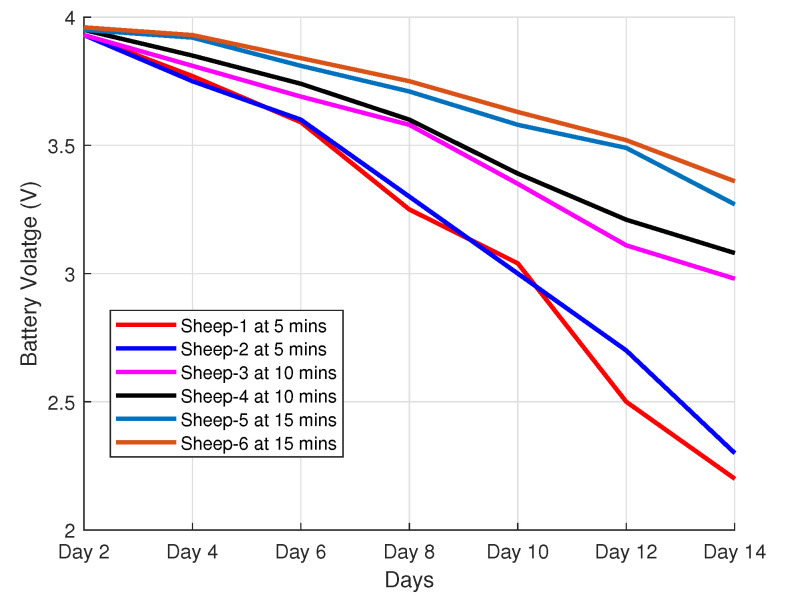
Battery discharge measurement.

We present, in Figure 11, the battery discharge of the devices over a 14-day period with respect to the sampling intervals of 5 min, 10 min, and 15 min in an ADR-enabled scheme. Six devices are evaluated and divided into three different categories, with each category sending its information at different sampling intervals, as shown in Table 3. The full discharge test was performed on a fully charged battery. From the figure, we observe that sampling at 15 min reduces the discharge rate as compared to 5 min and 10 min. In addition, the battery lasted for all devices operating at different sampling times during the 14-day experiment. The device software is configured in such a way that when the battery level drops beyond 2.8 V, the GPS module will not be able to obtain a GPS fix. Based on this observation, the GPS modules of the devices transmitting at 5 min intervals are disabled at day 10 of the 14-day period. To minimize the energy consumption, the end device’s software is designed to enter low-power modes whenever there are no active functions, as described in Section 3.2.

### 4.6. Localization Accuracy

The accuracy findings of the flock of sheep through GPS are shown in this subsection. Each device obtains the location data from the satellite in a periodic manner. In our experiment, we set the sampling period for all devices to be 5 min. Table 4 presents the results of a 7-day field test. MD represents the device’s maximum distance from the sheep area’s perimeter. The average distance (AD) is denoted as the mean distance of all Sheep-i outside of the sheep area, while out of bounds (OB) is defined as the percentage of sheep that are located outside of the sheep area. We can make the following observations: In a 7-day experiment, the percentage of sheep outside of the sheep area sampling at 5 min was quite low, showing good accuracy. This answer, however, does not offer the exact position of the flock of sheep, but rather the general area of where the flock of sheep is located. Furthermore, outside of the sheep region, the flock of sheep has an average distance of less than 5 m from the boundary, demonstrating the accuracy of the localization procedure. Lastly, we also notice that Sheep-8 achieved the maximum distance of 14.2 m from the boundary, indicating some discrepancies in the localization process. This could be due to a shorter mission life to retrieve a reliable valid GPS signal, resulting in out-of-bounds signals.

### 4.7. Average Delay

**Figure 12:**
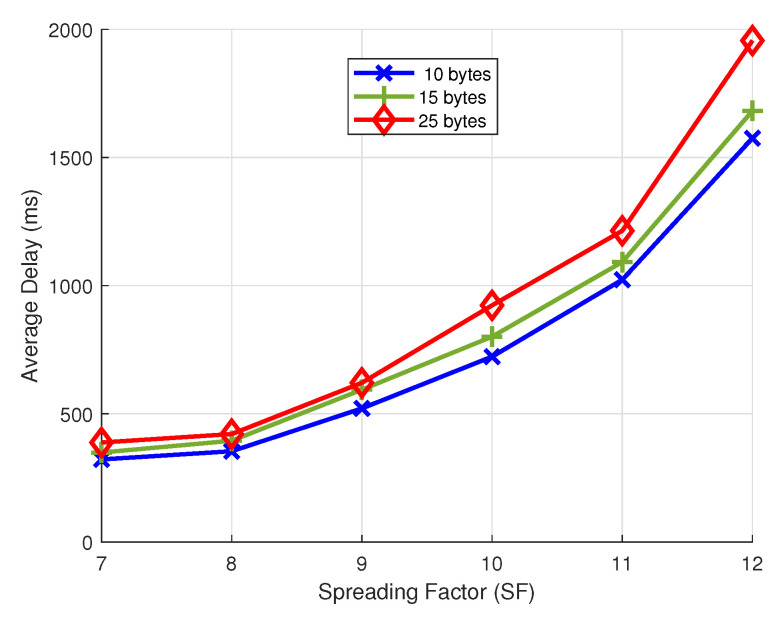
Average delay.

In this subsection, we demonstrate the average delay for different payload sizes while varying the SF. The average delay per received uplink packet is calculated by dividing the total delay of all the received uplink packets by the number of received uplink packets. The delay of one uplink packet is the time from when the uplink packet is first transmitted until it is successfully received at the LoRaWAN gateway and becomes available to the network server. We consider three payload sizes: (a) 10 bytes, (b) 15 bytes, and (c) 25 bytes. These payload sizes are chosen based on the information we want to send, and also following the maximum allowed payload size of 51 bytes according to the specification when using slow transmissions with SF 12. We consider 10 end devices sending their uplink packets to the network with a 15 min sampling interval for a duration of 7 days. Our results in Figure 12 show an average delay of 10 bytes per payload packet, which increases from 322 ms at SF7 up to 1575 ms at SF12. While the values at SF7 to SF11 for the different payload sizes are comparatively low, we observe a considerably spread at SF12, resulting in a delay of up to 1956 ms for a payload size of 25 bytes. Our results follow the relationship between the time on air (ToA) and the SF. For higher SF, the impact of the payload size on ToA increases.

### 4.8. Collisions

**Figure 13:**
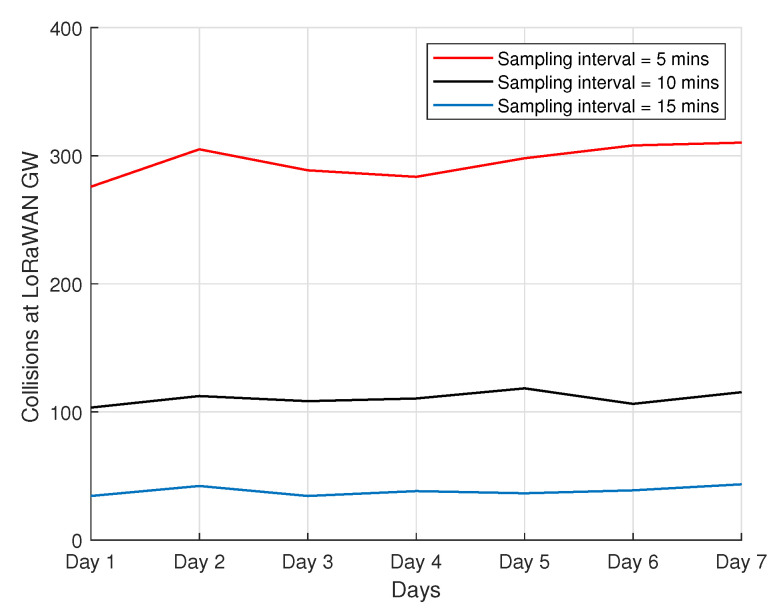
Collisions perceived by LoRa gateway.

In this subsection, we analyze and comment on the number of perceived collisions concerning the gateway. The result in Figure 13 shows the collisions at the gateway when considering three different sampling intervals in a 7-day period. Although the number of nodes is relatively small, we were still able to observe some collisions at the network server when varying the packet frequency. The experimental results show that there is a noticeable reduction in collisions when the sampling interval is 15 min, compared to the high packet frequency (every 5 min). When the packet frequency is low (every 15 min), this mechanism helps in providing a better energy usage, a higher reception throughput, and fewer collisions.

## 5. Discussion

The main goal of this field test is the presentation of GPS for localization and using LoRaWAN for control and monitoring. Modern telecommunications are aimed to provide solutions that are carefully tailored to the specific requirements of the domain. The motivations of our real trial were related to the possibility to control a flock of sheep while also providing appropriate warning to the companies that typically leave hundreds of animals grazing. The collected data will be processed in the cloud to extract relevant information related to the behavior of a single sheep and the flock itself. Many aspects were covered in this interdisciplinary collaboration that was designed not just as a demo, but as a field test, as well as highlighting the specific requirements that qualified the challenge of this future service aimed at improving the quality of life of the operators in the domain and of the animals themselves, considering situations such as car crushes, train accidents, etc.

An LoRaWAN coverage of one of Italy’s most important veterinary campuses was achieved, along with the establishment of a platform that will be extended with future services enabled on the same infrastructure. Many issues, such as the frequency of data acquisition, event-driven (for example, based on accelerations or chance of position) or periodic transmission of data, setup of LoRaWAN parameters, localization precision, power consumption, asset management, ease of use on field by the operating staff, coverage, and lifetime, were considered in the framework of this real experience, starting with a limited number of devices to better maintain control of the many different aspects ranging from periodic recharging of the devices to the visualization of the data. The experience also gave some relevant feedback on the adoption of LoRaWAN in this application domain.

Field tests show that the average PDR in both ADR-enabled and ADR-disabled schemes was over 82.5%, which is efficient when considering a network with these characteristics. From the results given, we can conclude that ADR-disabled networks behave better than ADR-enabled networks with a small number of end devices when considering the packet delivery ratio. We also observe, based on the results, that the use of ADR in LoRa networks has a noticeably positive impact on the energy consumption of the devices in the network, but comes at the cost of the slow convergence of the ADR-enabled scheme to adapt to the changing link conditions, requiring days to converge to energy-efficient communication state. The importance of the ADR mechanism for dynamic and scalable LoRa networks was demonstrated in our research. Despite the fact that the number of nodes required to fully explore the potentials of ADR is quite minimal, our investigations revealed certain variations when ADR is enabled. Our future research will try to expand the pilot study of a small-scale scenario to a larger network with hundreds of nodes, several gateways, and kilometers of areas covered, demonstrating the impact of ADR on scaling and deployment capacity.

When compared to cellular connectivity, the usage of LoRa for communication consumes less energy. With minimum infrastructure, our experimental results show great coverage of the site area. For low data rate demands such as location reports, LoRaWAN configurations have been shown to be cheaper and more convenient than deploying cellular repeaters. Even if the requirements include motion reports with the accelerometer data, our system proves reliable at the expense of extra battery life and bandwidth usage. LoRaWAN also ensures reduced radio-frequency radiation to the animals, which means less heating due to SAR (specific absorption rate). Such power is completely safe, as described in [77]. Other LPWAN technologies, such as Sigfox, use a similar amount of energy, but duty policies allow just 144 messages per day, which is not sufficient when sending messages every 5 min. Two-way satellite communications’ main drawback is associated with power consumption and operation costs (which is quite high if we are sending movement data), and other wireless technologies, such as Zigbee, are not adequate due to higher power consumption, higher difficulties in the management and setup of the infrastructure, and increased problems with respect to scalability.

A lesson learned from this work is regarding the methodology to follow for the design and development of the hardware and software components for the location monitoring system of our livestock in real time. One decade ago, Langendoen et al. [78] wrote a foundational paper listing everything that went wrong in a precision agricultural deployment similar to our use case, which includes board failure, unreliable network communication, high power consumption, etc. In our deployment, low-power devices for IoT applications have substantially evolved and have radically changed the suitability of embedded devices on the field in around one decade. However, although IoT technology has successfully transitioned from the academic to the commercial world, when uncommon use cases are considered, the development and deployment of applications still require accurate customization and complex integration activities, also giving the possibility to detail some of the specific requirements in the domain.

## 6. Conclusions

This paper has presented a solution for dealing with this problem of lack of established communication infrastructures for controlling the movements of animals from extensive livestock farms. This study describes the development of a Smartsheep location monitoring system based on integration of ad hoc IoT devices, LoRaWAN technology, and cloud computing. The developed system consists of three main parts: (i) the ad hoc IoT device in the form of a wearable that is equipped with inertial sensors, GPS, and an LoRaWAN transceiver to transmit data retrieved from the sensors to the LoRaWAN network, (ii) an LoRaWAN-based communication network that collects data retrieved from the sensors and transmits it to the cloud, and (iii), the cloud solution that ensures a reliable, robust, and secure infrastructure that supports automatic scaling of resources according to the system needs.

To experimentally evaluate the location monitoring system, the system was deployed in the northern part of Italy as a pilot study, evaluating its performance in terms of the network reliability, location accuracy, battery discharge measurement, energy consumption, and delay. Based on the results, the synergy between LoRaWAN technology and GPS technology provided a satisfactory compromise between accuracy, reliability, and energy consumption. In addition, ADR-enabled schemes were compared with ADR-disabled schemes, showing the effect of ADR on energy consumption and packet delivery ratio. This study presented a method to locate and monitor livestock in extensive farming, which also includes the necessity to track the movement of the animals to evaluate their impact on the plant biodiversity, as well as the intention to virtually reduce the distance between the animals and the shepherds.

This work, motivated by the Smartsheep project, will conduct future tests that can be carried out in the mountainous pastures and at a large scale to study the impact of the environment (in terms of high temperature, air humidity, pressure, rainfall, etc.) on the LoRa performance, and propose a suitable propagation model for mountainous pastures from the obtained results.

## Figures and Tables

**Table 1:** Pros and cons of different localization techniques.

Localization Techniques	Advantages	Disadvantages
ToA	It can provide high localization accuracy. It does not require fingerprinting. Low attenuation.	It requires time synchronization between the transmitters and receivers. It might also require time stamps and multiple antennas at the transmitter and receiver. Line of sight is a requisite to achieve good accurate position.
TDoA	It ensures low latency and high performance reliability while processing thousands of received blinks. It can provide high localization accuracy. It does not require fingerprinting. It does not require clock synchronization among the device and the reference node. Low attenuation.	It requires clock synchronization among the reference nodes. It might also require time stamps. Large bandwidth is also a requirement. Localization accuracy depends on the signal bandwidth, sampling rate at the receiver, and the existence of direct line of sight between the transmitters and the receiver.
RSSI	It is cost-efficient. It is easy to implement. It is compatible with the majority of the technologies. Low hardware requirements.	It is very sensitive to interference, noise, and multi-path fading. It can require fingerprinting. Lower accuracy.
AoA	It can provide high localization accuracy. It does not require fingerprinting.	It might require directional antennas and complex hardware. It is not cost-efficient. It also requires complex algorithms. Performance deteriorates with increase in distance between the transmitter and receiver.

**Table 2:** A summary of related works.

Ref.	Target Animal	Localization Technologies	Localization Method	Cloud Infrastructure	Nature of Research
[41]	Cow	BLE	RSSI	NS	Performance Analysis
[44]	Cattle	Zigbee	ratiometric vector iteration (RVI)	NS	Performance Analysis
[45]	Cattle	Zigbee	NS	NS	Use Case Analysis
[53]	Cattle	GPS + LoRaWAN	NS	Yes	Laboratory and Field Tests
[54]	Cattle	GPS + LoRa	RSSI	No	Performance Analysis
[55]	Cattle	NB-IoT	NS	Yes	Performance Analysis
[13]	Sheep	NS	RSSI	Yes	Performance Analysis
[70]	Goat	GPS + Bluetooth, LTE	NS	NS	NS
[56]	Cattle	GPS + Sigfox	NS	NS	Performance Analysis
[46]	Cattle	GPS + GSM	NS	No	Statistical Analysis
[52]	Cattle	Zigbee	ToA	No	Experimental Analysis
[48]	Cattle	GPS + LoRa	NS	No	Field tests
[61,62]	Cattle & Sheep	GPS + UAV	NS	No	Simulation tests

NS: Not specified.

**Table 3:** Sampling intervals.

End Devices	Sampling Interval
Sheep-1	5 min
Sheep-2	
Sheep-3	10 min
Sheep-4	
Sheep-5	15 min
Sheep-6	

**Table 4:** Location accuracy.

*Sheep-I*	AD (m)	MD (m)	OB (Percentage)
Sheep-1	3.5	5.4	3.16
Sheep-2	4.98	7.6	1.32
Sheep-3	2.1	3.2	0.82
Sheep-4	0.32	0.32	0.056
Sheep-5	0.85	1.1	0.12
Sheep-6	2.7	3.9	1.82
Sheep-7	4.78	14.2	2.52
Sheep-8	1.5	1.5	0.64
Sheep-9	3.4	5.4	1.64
Sheep-10	2.52	4.3	0.76

## Data Availability

Not applicable.
